# Factors Associated With Digital Confidence and Use of Technology Among Older Queenslanders

**DOI:** 10.1111/ajag.70117

**Published:** 2025-12-01

**Authors:** Jeni Warburton, Mehak Oberai, Connor Forbes, Zhiwei Xu, Aaron Bach, Ella Jackman, Sarah Cunningham, Sebastian Binnewies, Shannon Rutherford, Steven Baker

**Affiliations:** 1 La Trobe University, Melbourne, Victoria, Australia; 2 School of Medicine and Dentistry, Griffith University, Gold Coast, Queensland, Australia; 3 School of Information and Communication Technology, Griffith University, Gold Coast, Queensland, Australia; 4 Griffith Institute for Human and Environmental Resilience (GIHER), Griffith University, Nathan, Queensland, Australia; 5 School of Health Sciences and Social Work, Griffith University, Gold Coast, Queensland, Australia; 6 University of Queensland, Brisbane, Queensland, Australia

**Keywords:** aged, digital divide, surveys and questionnaires, technology

## Abstract

**Objective:**

Digital technology increasingly plays an important role in supporting older adults to live longer and healthier lives. However, research consistently identifies barriers to technology usage for some older adults, particularly as they age. This study draws on data from a survey of older adults (aged 65 years and older) to improve our understanding of the role that confidence with technology plays in older adults’ technology acceptance.

**Methods:**

We analysed technology usage and acceptance data from 547 Queensland older adults (53% male, 47% female) gathered as part of a larger survey examining how technology may assist the development of an in-home heat early warning system for older Queenslanders. Respondents fell within five age range groups, 65–69 years (24%), 70–74 years (29%), 75–79 years (28%), 80–84 years (12%) and 85+ (7%).

**Results:**

Respondents from the highest age range groups were more apprehensive about, and were slower to adopt, new technologies. Moreover, overall use of technology also declined with age, implying the need to consider ageing cohorts when considering technology acceptance. When looking at digital confidence, our results highlight that age was the only independent variable that predicted (inversely) digital confidence, and somewhat counterintuitively, frustration with technology. This may suggest that older cohorts avoid using new technology to avoid the risk of frustration.

**Conclusions:**

Results from the survey data add to our knowledge of the patterns of technology usage and acceptance by older adults. Our analysis underscores the need to consider variables such as confidence or frustration with technology when considering whether cutting-edge technologies will benefit older users.

## 1 Introduction

Globally, some of the most significant social trends in recent years include the ageing of the population and the growth of digital technologies [1–3]. This duality has led to attention to the concept of gerontechnology, [4] where technologies have the potential to support older people to live longer and healthier lives [5] and facilitate ageing in place through interventions such as smart housing, health monitoring, e-health and social care [4, 6–7]. Technology has the capacity to improve the quality of life for older people [8] by assisting to combat social isolation [9] and improve mental health [10]. Technological interventions also have the capacity to assist older people to respond to the challenges posed by climate change, where they may be more vulnerable to factors such as natural disasters or heat stress [11, 12].

Thus, technology has the potential to have a positive effect on older people, and to confer practical, physical, social and emotional benefits, enabling them to age well [13]. However, the literature talks overwhelmingly of a digital divide, whereby older people are less accepting of technology than younger people [8, 14–15]. Results from an Australian study developing a digital index, for example, showed that digital ability declines with age [16]. In particular, older adults frequently use well-established technologies but are much slower to adopt emerging technology [8, 17].

Technology acceptance by older adults is a complex issue affected by multiple factors, [4, 18] including attitudinal, functional or physical dimensions [1]. Gitlow [18] suggests that while age-related changes such as vision or motor skills may impact usage, attitudes towards technology are another important dimension. Several models have been developed to assess factors relating to the cost, ease of use and usability of technology such as the technology acceptance model (TAM) [19] and the unified theory of acceptance and use of technology (UTAUT) [15, 20]. Chen and Chan [21] have also adapted the TAM to specifically consider older adults’ acceptance of everyday technology, and the senior technology acceptance model (STAM) has been widely used, often in modified versions since its introduction [4].

Digital confidence is an important aspect of technology acceptance and relates to an individual’s emotional and psychological readiness to engage with technologies, particularly their belief in their ability to use technology effectively and independently. It is this emotional aspect that separates digital confidence from related concepts, such as digital skills, which relate more to technical abilities, and digital literacy, which relates more to the ability to critically understand and evaluate the use of digital content [4, 22]. Likewise, a lack of digital confidence is also widely seen as a core barrier to the use of technology in later life [14, 22]. There is some evidence to suggest that confidence can be impacted by negative stereotypes associated with age, [2] and reinforced by a lack of opportunities to learn how to use technology [6]. For example, surveys of older technology users in the United States have demonstrated that confidence is a key challenge and that many are not confident in their ability to learn and get the best out of new technology [23].

Although a lack of digital confidence can be identified as a core barrier to the use of technologies, there is far less literature on what factors impact confidence or who is most at risk. Thus, Schroeder et al. [5] in their extensive international review of factors associated with older adults’ use of digital technologies, note that despite the significance of the topic, research is surprisingly limited. Furthermore, the review found only one Australian study of some 58 studies reviewed, a finding also supported by a more recent review that found only two Australian studies out of 83 articles reviewed [15]. This suggests a particular absence of Australian research in this field.

A related issue is that much of the gerontechnology literature lacks detail on how older adults’ attitudes towards technology use differ [7, 24] across the older age range [2]. Most studies focus on older people as one group aged 65 years and older [5]. This includes Australian reports; for example, a report by National Seniors discusses ‘comfort with digital technologies’ for older people but provides no comparison across age groups [25]. Overall, extant literature overwhelmingly fails to consider the diverse life and work experiences across the life course, and how this impacts older adults’ capacity to learn new skills and build confidence in using new technologies [3].

Thus, the digital divide associated with ageing and the potential lack of digital confidence among older adults are significant issues in the technological age. Due to the substantial gaps in knowledge around this topic, particularly in the Australian context, this paper aimed to contribute to understanding older people’s attitudes towards digital technologies. Specifically, we draw on data from a large Queensland study to explore the following research question: *What factors influence the digital confidence of older Queenslanders?*

## 2 Methods

The paper draws on data from a large omnibus survey designed to understand how technology can assist the development of an in-home heat early warning system for older and potentially at-risk Queenslanders [26]. A 144-question survey was developed to gather information on heat wave risk, knowledge and behaviours, as well as digital literacy of older people. For details on survey design, validation, conduct, data cleaning and incomplete data, please refer to [26]. This previous paper contains a more extensive discussion of the design and sample characteristics of the data that we draw on in this paper.

An important preliminary stage of this study was assessing older people’s views on their use of technology, and particularly their capacity to accept new technologies. The concept of digital confidence among this demographic was therefore central to the research, and only survey elements directly relevant to the research question and participants’ views on technology are reported in this paper. These questions were drawn from the senior technology acceptance model (STAM) ([27]. Ethos Survey 2022–Technical Report. The Ethos Project, Griffith University. Gold Coast, Queensland, Australia) (Section K: Questions K1, K2, K4, K5, K6, K7, K8, K10, K11, K12, K13, K15 and K18). To measure digital confidence, Question K10 asked: ‘How confident are you in using computers, smartphones, or other electronic devices to do the things you need to do online?’ Responses were recorded on a Likert scale ranging from *Don’t know* to *Very confident*. In addition, Question K18.1—’I feel frustrated using digital services’ (Agree/Disagree)—was used to assess levels of frustration experienced by participants when engaging with digital platforms. Completion of the survey was considered as providing consent to use the data provided in a deidentified form.

The study protocol and ethics documents were approved by the Griffith University Human Research Ethics Committee (2022/627). The target population was those aged 65 years and older living in Queensland. The estimated size of the older population was assessed at around 850,000, and thus, a sample size of at least 384 was required for the results to meet a 95% confidence level with a 5% margin of error. Participants were recruited via stratified and convenience sampling methods through, first, an online panel, and then through extensive publicity (local media, newsletters, flyers and snowball sampling among researchers), respectively. A paper mode was also utilised to include those with limited access to the internet, specifically to ensure the inclusion of some of the older cohorts, and these were posted to recruited participants together with an information sheet. Online surveys were administered through Qualtrics (Qualtrics, Provo, UT). The online and paper-based responses were merged for data analysis, after analysis was conducted to ensure that there were no key differences in responses.

To ensure rigour in the study, key socio-demographic features of the sample were compared with overall Queensland data for this age group to verify the sample was broadly representative of the Queensland population [28].

### 2.1 Quantitative Analysis

The data analysis was carried out in SPSS (28.0), where descriptive and bivariate analyses were performed initially on the merged dataset. In bivariate analysis, *χ*^2^ or Fisher’s exact tests (expected cell frequencies less than or equal to five) were used to test for the inter-relationships among variables (as all were categorical variables) with a *p* value < 0.05 considered to be statistically significant.

Initially, the descriptive analysis of the digital technology related questions (K1, K2, K4, K5, K6, K7, K8, K10, K11, K12, K13, K15 and K18) (see questionnaire attached in the File S1) was carried out. This was further followed by a basic *χ*^2^ analysis to explore whether confidence in using technology was related to: (1) Sociodemographic factors such as age, gender, education, income, self-reported health and financial status, disability status or living alone, and (2) Frustration in technology usage. The variables that were found to be statistically significantly associated with the confidence in using technology (age and frustration in technology usage) were then fitted into a multinomial regression model (with outcome variables as categorical variables with more than two outputs) to further understand how various factors were associated with the confidence of using digital technology in older Queenslanders. Not at all confident was used as a reference category for the regression model.

Additionally, as frustration with using technology was related to confidence in using technology, basic *χ*^2^ analysis was carried out to explore whether the frustration in using technology was related to sociodemographic factors such as age, gender, education, income, self-reported health and financial status, disability status or living alone. Variables significantly associated with frustration (age and health status) were then entered into a generalised linear model with a binomial distribution (outcome variables with two outputs) and a logit link (i.e., logistic regression) to examine their adjusted association with the likelihood of feeling frustrated about using technology.

## 3 Results

A total sample size of 547 (complete responses) was achieved for the survey, with a mix of 447 online responses (412 complete) and 138 paper survey responses (135 complete) out of 201 mailed. The incomplete surveys were not included in the final dataset. As shown in Table 1 (and reported in [26]), the sample can be considered representative of older adults in Queensland based on comparison with existing population-based data (for age group, self-reported health and finance status, and gender ratio). However, the education attainment status of our sample was slightly higher than the total Queensland population.

Initial analysis of the digital section of the survey (from K1 to K18) revealed several key insights into older Queenslanders’ engagement with digital technologies. Smartphones were the most commonly used device (85%), followed by smart TVs (65%), laptops (59%) and tablets (55%). In contrast, wearables (2%) and standa-lone GPS systems (19%) were the least common. Application (App) usage patterns showed high engagement with social media (78%), virtual assistants (65%) and entertainment apps (65%), while health apps (14%) and news apps (46%) had lower uptake. Utility-based apps such as government services (68%) and banking apps (60%) were widely used. Frequency of app use was high, with over 51% using apps multiple times a day and 70% using them at least daily, while only 1% reported never using apps. Key barriers to app usage included privacy concerns (29%), lack of confidence (23%) and a perceived lack of need (21%), with 8% feeling too old to learn, highlighting age-related digital exclusion.

Although 79% felt confident using devices, only 59% felt confident learning new technologies. Learning preferences leaned towards printed manuals (39%) and web-based resources (31%), with small in-person groups (10%) preferred over large groups or video calls. Digital financial services were considered essential by many, with 60% relying on online banking and 58% on online payments. Despite this, 62% reported frustration with digital services, 64% cited cost as a barrier, and only 39% felt these services made life easier. Encouragingly, 58% expressed interest in more training, and 52% indicated they would use more digital services if supported. Finally, perceptions of technology’s impact were nuanced, with 49% viewing it as equally positive and negative, and 35% seeing it as mostly positive.

**Figure 1:**
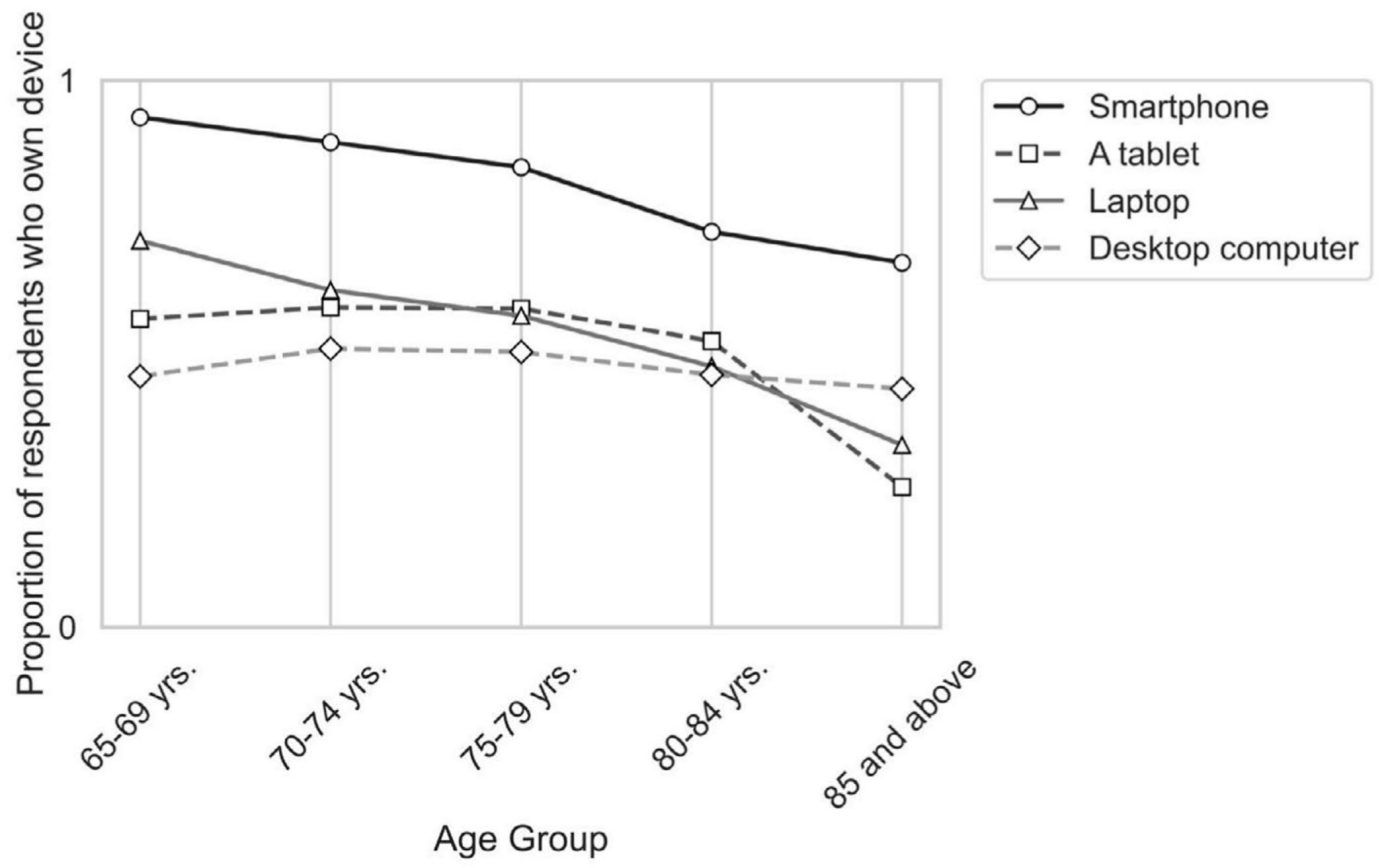
Proportion of device owners by age group.

Following the descriptive analysis, the next stage of analyses aimed to assess the sample’s existing use of technology, specifically, whether there were any differences by age. As stated earlier, respondents used a mix of smart phones, tablets, laptops and desktop computers, with smart phones (85%) being the most popular. However, there were some differences by age (Figure 1), with a decline in the use of all devices over age cohorts except for desktop computers, which remained less used overall but consistent by age.

**Figure 2:**
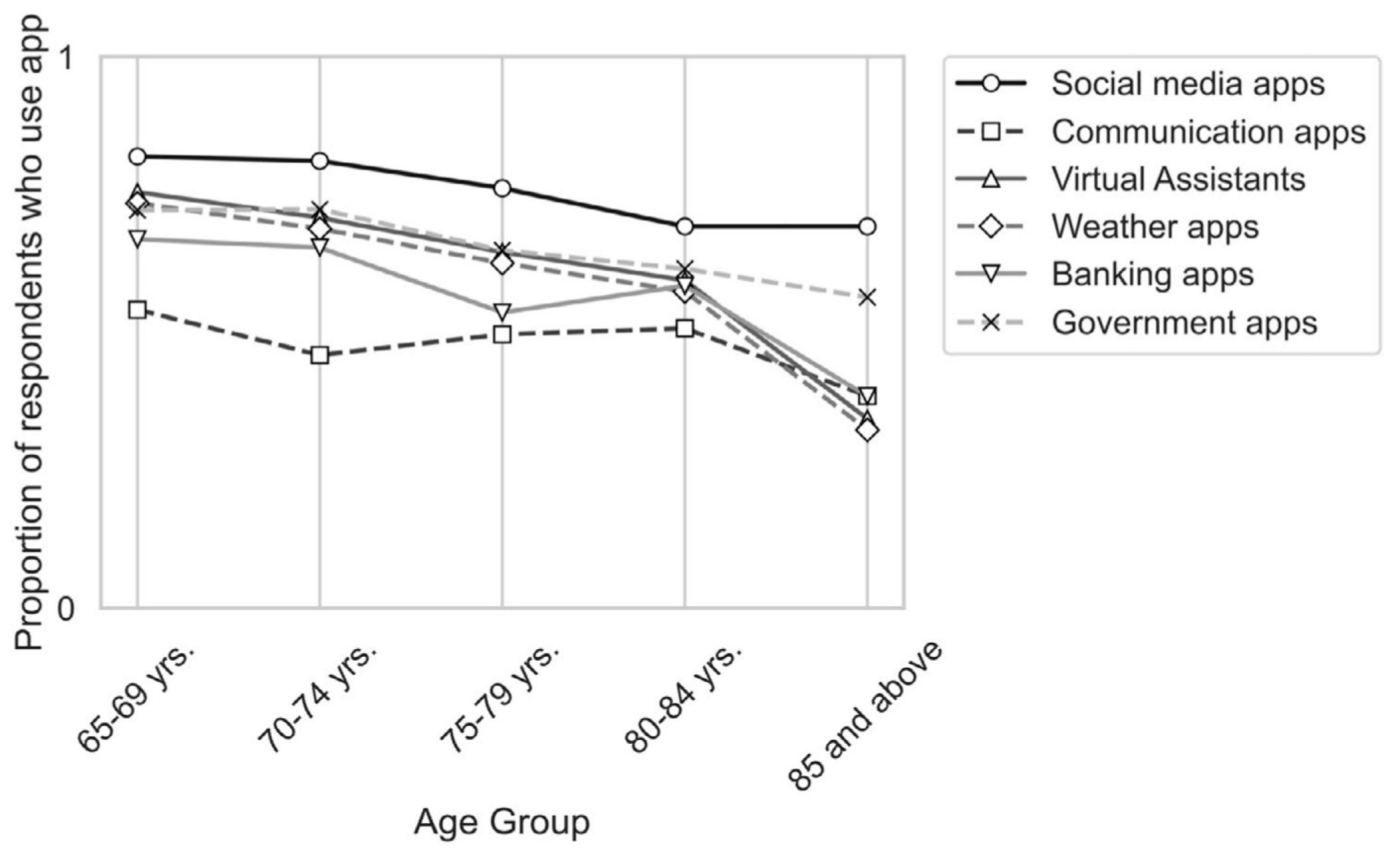
Proportion of respondents that use apps by age group.

**Figure 3:**
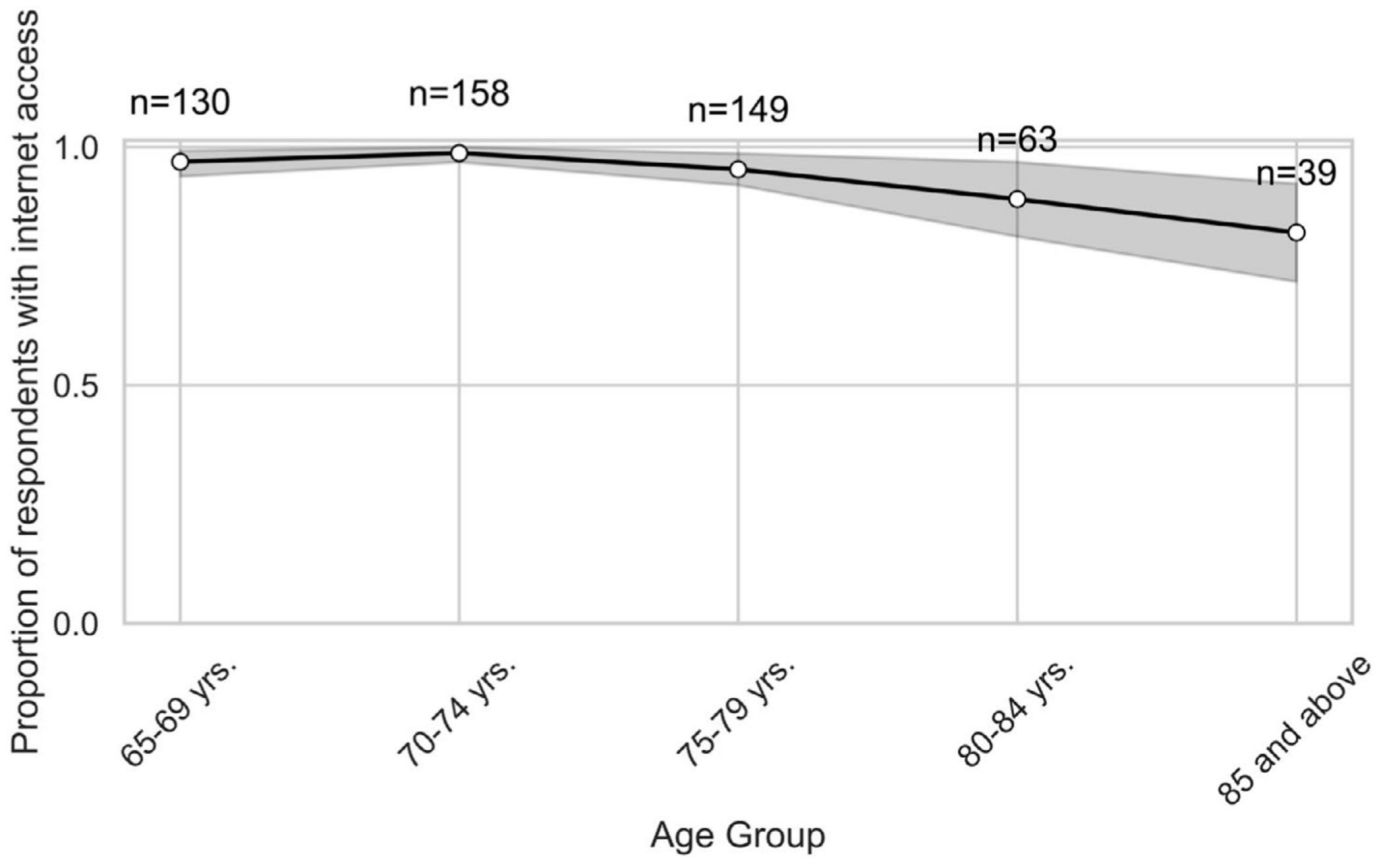
Proportion of respondents who report having internet access at their place of residence.

In looking at the apps used by participants, Figure 2 shows that while social media apps (78%) were the most common, other apps were also accessed by participants, with a notable decline by age cohort across all categories. Further, internet access was common across participants, with some decline in the over 80-year-old cohort (Figure 3).

**Figure 4:**
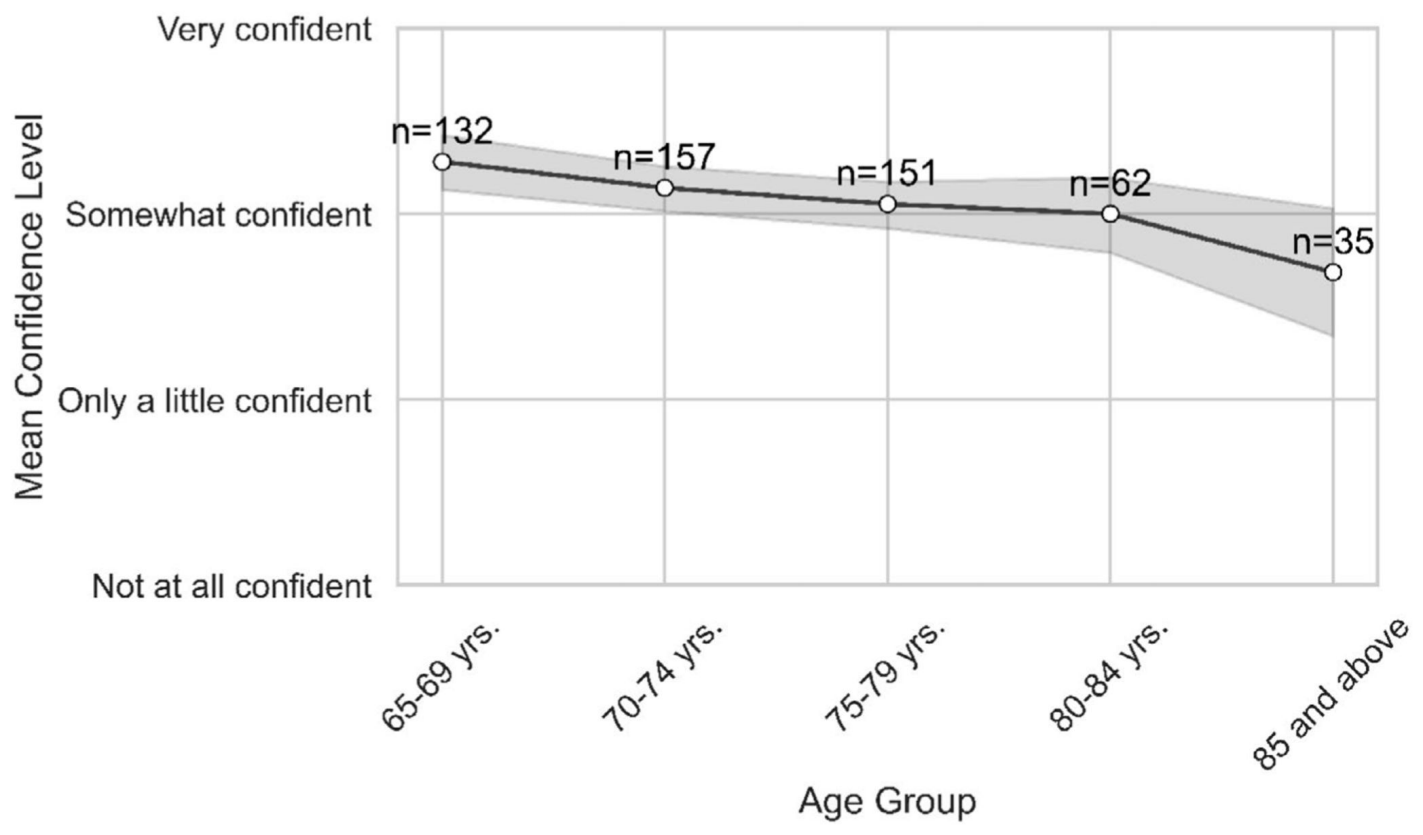
The mean technology confidence level of respondents by age group.

When asked whether they liked using technology (K1.1), there was only a moderately positive response across the scale, with most responses across all age groups close to the midpoint of the Likert scale (neither agree nor disagree). This unenthusiastic response along with the fact that most participants had some level of digital presence suggested that it was important to look at their levels of digital confidence in using these devices and apps and various factors, which might be impacting their experience with technology. However, Figure 4 shows that participants were reasonably confident, although there was a decline in digital confidence over the age cohorts.

After exploring the bivariate relationships between digital confidence and the suite of independent sociodemographic variables, multinomial regression analyses were conducted to further explore these relationships. These showed that a person aged 65–69 years was 6.2 times more likely to be confident in using technology than someone aged 85 years or above. While age was associated with other variables such as financial status, education level and living situation (but not gender, health or disability status), age was the only independent variable predicting confidence.

Respondents did, however, report they were 4.3 times more likely to feel confident if they were not frustrated with using technology. Similar results applied across these analyses in relation to using new forms of technology—thus, the older cohorts were less confident overall with technology.

A *χ*^2^ analysis revealed a significant association between feeling frustrated and age and health status. Further, generalised linear model analysis to investigate the relationship between feeling frustrated (binary variable) and the suite of independent variables showed that compared with people aged 65–69 years, those aged 75–79 years were 49% less likely to feel frustrated about using technology; and compared with those with poor self-reported health, those who had good health were 123% more likely to feel frustrated using technology. Other sociodemographic variables did not show any significant relationship with frustration. These results show that those in the younger age groups and those in good health were more likely to feel frustrated with using technology, even though younger age cohorts were more likely to feel confident with using technology.

## 4 Discussion

The results reported here are part of a broader study designed to explore older Australians’ acceptance of technology as a precursor to developing an in-home heatwave early warning system. The paper specifically addresses the concept of digital confidence, identified as key to older adults’ acceptance of existing and emerging technologies. Thus, the specific research question addressed in this paper was: *What factors influence the digital confidence of older Queenslanders?* While there is a growing literature around the role of digital technologies in helping older people live longer, healthier lives, [5, 15] far less is known about what underpins the age-based digital divide and how different cohorts of older people may experience digital confidence. Further, there is less research into this topic in Australia, yet ageing policies and practices vary across different contexts, suggesting a need to understand technology acceptance, and specifically digital confidence, from an Australian perspective. The present study aimed to explore these gaps in knowledge by providing insights into the digital engagement of older Queenslanders.

Initial results showed participants’ use of digital technologies across a range of devices and applications. Generally, this showed quite strong usage indicating high digital engagement in older adults, but in both cases, a decline by age cohort. Thus, younger age cohorts were more likely than older cohorts to use smart phones, laptops or tablets, with only traditional desktop computers being used by all cohorts. Similarly, while all cohorts use social media applications, older cohorts were increasingly less likely to use other apps such as communication or banking apps. Yet, internet access was fairly uniform except for the oldest age group. These results all suggest that older cohorts use a less diverse mix of both technologies and applications and that their use of technology overall declines with age. Additionally, the decrease in confidence (79%–59%) in learning new technologies suggests a gap in the digital adaptability among the older adults for unfamiliar or evolving technologies. This gap in adaptability is also highlighted by the low usage of new or more specialised technologies such as wearables and health apps.

Our results are somewhat consistent with the literature, which highlights that older people are more likely to be apprehensive about technology [14] and are slower to adopt new technologies [8]. However, it signals the potential importance of looking at different older age cohorts, as this may well not be true of younger cohorts of older adults, who may have had, for example, more recent experience with technology in the workforce. The results in the present study suggest that stereotypically viewing all older people as homogeneous and lagging behind in technology may be inaccurate, as some authors propose [2]. A recent meta-analysis of research conducted using various models of technology acceptance (TAM, UTAUT, STAM) has noted that most of the research looking at age-related factors has tended to classify participants into young and old [29]. While our research supports the view that a more complete understanding of older adults’ confidence in using technology can be gleaned from a more granular approach that considers age cohorts, we also note that measuring other indicators such as functional, subjective or psychosocial age may also lead to a better understanding of technology acceptance and confidence [29].

It is important, therefore, to consider a lack of digital confidence as a potential barrier to use. This is particularly relevant as findings show that most respondents were only moderately positive about technology use, raising the question as to why this is the case when the literature highlights the importance of digital technology to healthy and active ageing [9]. Findings from the present study showed that the younger cohort was significantly more confident in using technology, including learning new technologies, with an overall decline across cohorts. Interestingly, age was the only independent variable predicting digital confidence, despite the literature highlighting other factors such as gender or income [8, 30]. These findings are a particular concern given new and cutting-edge technologies are routinely put forward as cost-effective solutions to age-related issues and research consistently demonstrates that those in the much older groups are in most need of self-management for chronic care [6, 17]. Our findings therefore highlight the need for careful consideration of factors relating to technology confidence in older cohorts when developing gerontechnology.

It may be that frustration with technology is also related to digital confidence [24]. However, in the present study, findings showed that those who are more likely to use and embrace new technologies (younger cohorts and those in good health) are more likely to feel frustrated with it. This is an interesting result, somewhat counter-intuitive at first glance. One potential explanation is that this result is yet another dimension of the more conservative approach to technology use of those in the older cohorts as demonstrated in our results. In this hypothesis, as older cohorts experience greater frustration, they simply decide not to use more diverse technologies and applications. Indeed, some authors have suggested that older adults may become frustrated that their existing skills do not apply to new technologies [3, 17]. While only one potential explanation for our findings, we believe this hypothesis is worthy of further investigation in future studies, particularly as new technologies develop in the ageing space.

Key strengths of the current study were its focus on digital confidence as a key concept and use of existing measures to test this concept. Further, the study utilised a large sample across the age range, which is broadly representative of the Queensland population. However, there are also important limitations to note that could impact the generalisability of the results. First, the majority of respondents to the survey did so online (447 of the 547 completed surveys that were included for analysis), which may have led to a bias towards increasing confidence in using technology. Second, it is important to note that the survey reported in this paper was cross-sectional, not longitudinal. We therefore cannot state whether the age-related differences are due to age itself or are rather due to the context of ageing. As new cohorts age, it may be that older people are more used to new technologies, and hence more confident. However, it should be noted that, as our study shows, they may also become frustrated with having to learn new techniques. Thus, it is uncertain whether age-related differences will persist over time and further research is warranted to account for this dimension of the study over time.

Specifically, the present study has shown differences across age cohorts in relation to technology use and digital confidence. This has not generally been reflected in prior research, yet it is important to consider, particularly as there may need to be different solutions required in terms of involving older people in the research and design of technologies; [2] building confidence through positive experiences [22] and more targeted classes [3]. These all need to be adapted to older adults’ stage of life, with more research conducted to explore how diverse older people can gain the relevant skills they need to become proficient technology users.

## 5 Conclusions

Results from the present study suggest that age impacts digital confidence and that frustration with digital technology also impacts older users’ willingness to adopt new and emerging technologies. This paper has the potential to contribute to our knowledge in this critical field of ageing and technology, particularly focussed on the different experiences of cohorts of older people. Given the growing role of digital technologies in an ageing society, it is essential to understand how older adults engage with these tools and what influences their confidence in doing so. This study highlights the importance of tailoring technology interventions to the diverse needs and experiences of older cohorts. Rather than assuming uniformity across age groups, developers and practitioners should consider factors such as digital confidence, perceived usefulness and potential frustration. These considerations are critical to ensuring that technologies are not only accessible but also well-suited to the users they are intended to support. Further research is needed to deepen our understanding of how these dynamics evolve across different stages of ageing and to inform more inclusive and effective digital health strategies.

## Supplementary Material

S1

## Figures and Tables

**Table 1:** Comparative analysis of Ethos sample and wider Queensland (QLD) population demographics and education levels.

Variable	% Sample		% QLD population	
Age group				
65–69	24		30	
70–74	29		28	
75–79	28		19	
80–84	12		12	
85+	7		11	
Self-reported financial status				
Struggling financially	17		17	
Doing okay	47		47	
Comfortable	33		30	
Financially well off	3		6	
CALD representation	30		29	
Gender				
Male to female ratio	53:47		49:51	
**Education level**	**65–74**	**75+**	**65–74**	**75+**
Bachelor and above	24	27	15	9
Certificate III, IV, diploma	38	33	27	22
Year 10 and above or	36	35	39	36
certificate I/II				
Year 9 and below secondary	2	5	19	33

Abbreviation: CALD, Culturally and Linguistically Diverse.

## Data Availability

The data that support the findings of this study are available upon request from the corresponding author. The data are not publicly available due to privacy or ethical restrictions.

## Practice Impact

This study highlights the importance of considering different stages of ageing when implementing technology-based solutions. Results show older adults may choose to avoid cutting edge technology as they age due to the risk of frustration. Practitioners should carefully consider the age of their target audience when considering technology-based interventions.

## Policy Impact

Technology has undoubted benefits for supporting healthy ageing. However, our study suggests that policy makers should carefully consider the age group that any technology intervention is targeting, as confidence with technology is highly dependent on the age of the target audience.
